# Differences in residual lesion detection after treatment of metastatic prostate cancer based on dual-tracer PET/CT

**DOI:** 10.3389/fonc.2026.1713930

**Published:** 2026-03-12

**Authors:** Tengfei Li, Jianying Ma, Yaning Wang, Fei Li

**Affiliations:** Department of Computed Tomography (CT) Imaging, Nanyang Central Hospital, Nanyang, Henan, China

**Keywords:** ^18^F-FDG, ^68^Ga-PSMA-11, PET/CT, prostate cancer, residual disease

## Abstract

**Background:**

^18^F-fluorodeoxyglucose (^18^F-FDG) positron-emission tomography/computed tomography (PET/CT) and ^68^Ga-prostate-specific membrane antigen (^68^Ga-PSMA) PET/CT are widely used imaging modalities for the diagnosis and management of prostate cancer. However, comparative data on their performance in evaluating residual disease in treated metastatic prostate cancer remain limited. This study aimed to compare the efficacy of ^68^Ga-PSMA-11 and ^18^F-FDG PET/CT in detecting residual lesions after therapy in patients with metastatic prostate cancer.

**Methods:**

We retrospectively analyzed 26 metastatic prostate cancer patients who underwent both ^68^Ga-PSMA-11 PET/CT and ^18^F-FDG PET/CT at Nanyang Central Hospital between January 2023 and June 2025. A composite reference standard incorporating histopathology (when available) and follow-up contrast-enhanced CT or MRI was used to confirm metastatic lesions. Lesion-based detection rates and tumor-to-background ratios (TBR) were compared between the two tracers.

**Results:**

In post-treatment primary tumors, detection rates were 92% for ^18^F-FDG and 100% for ^68^Ga-PSMA-11. ^68^Ga-PSMA-11 demonstrated significantly higher SUVmax (median 17.08 vs 5.05, IQR 10.76-24.67 vs 3.90-6.38; P < 0.001, r = 0.81) and TBR (median 26.98 vs 4.81, IQR 17.00-38.97 vs 3.72-6.08; P < 0.001, r = 0.85) than ^18^F-FDG. For bone metastases, detection rates were 95% and 100%, respectively, with ^68^Ga-PSMA-11 again exhibiting superior SUVmax and TBR (8.29 ± 5.02 vs 33.77 ± 23.51, P < 0.001, d = 0.95; 6.56 ± 3.97 vs 33.08 ± 23.03, P < 0.001, d = 1.03). The per-lesion detection rate for lymph node metastases was 91% for ^18^F-FDG PET/CT and 96% for ^68^Ga-PSMA-11 PET/CT. SUVmax and TBR were significantly higher with ^68^Ga-PSMA-11 (17.02 (9.40-27.07) vs 4.17 (3.55-5.17), P = 0.005, r = 0.80; 26.88 (14.85-42.77) vs 3.96 (3.38-4.93), P = 0.002, r = 0.85).

**Conclusions:**

^68^Ga-PSMA-11 PET/CT shows higher tracer avidity and tumor-to-background contrast than ^18^F-FDG PET/CT for detecting residual primary tumors, lymph node, and bone metastases after therapy in metastatic prostate cancer. ^18^F-FDG PET/CT may serve as a complementary modality, and combined use of both tracers could enhance the detection of residual disease in treated patients.

## Introduction

Prostate cancer (PCa) is the second most frequently diagnosed malignancy in men. According to the latest estimates from the International Agency for Research on Cancer, approximately 1.5 million new cases and 397000 deaths were recorded worldwide in 2022, ranking PCa as the fifth leading cause of cancer-related mortality among males (1). While localized disease is associated with a 5-year relative survival rate of 96.8%, this figure drops to approximately 30% in patients presenting with distant metastases at initial diagnosis (2). The clinical course of PCa is highly heterogeneous and androgen-dependent, ranging from indolent to aggressively lethal phenotypes. Patients with high-grade, metastatic disease constitute the principal driver of prostate-cancer-specific mortality (3, 4).

Accurate staging is pivotal for tailoring individualized therapy and critically relies on imaging to detect nodal and osseous spread. Conventional cross-sectional imaging, however, still assesses nodal involvement based on size, morphology, and enhancement criteria, resulting in suboptimal diagnostic accuracy (5, 6).

Positron-emission tomography/computed tomography (PET/CT) integrates anatomical, functional and molecular information and has become indispensable for oncologic staging and restaging. A radiolabeled tracer accumulates preferentially in malignant tissue, thereby distinguishing tumor from background. ^18^F-fluorodeoxyglucose (^18^F-FDG), a glucose analogue that exploits the Warburg effect, is the most widely employed PET radiopharmaceutical and has been extensively evaluated in primary staging of PCa (7). Nevertheless, ^18^F-FDG PET/CT is hampered by limited tumor-to-background contrast in the prostate bed, physiologic urinary activity and a substantial rate of false-positive lymph node uptake, all of which curtail its specificity (8). The diagnostic utility of ^18^F-FDG PET/CT in prostate cancer is fundamentally constrained by the distinct biological characteristics of this malignancy (9, 10). Unlike many other solid tumors, localized prostate cancer typically exhibits indolent biological behavior with relatively modest glucose metabolic activity, resulting in limited ^18^F-FDG avidity and poor tumor-to-background contrast against adjacent normal prostatic tissue or benign conditions such as prostatic hyperplasia, thereby substantially compromising sensitivity for primary lesion detection (11). Moreover, the accuracy of ^18^F-FDG PET/CT in evaluating lymph node involvement and distant metastasis remains suboptimal (12, 13). Although ^18^F-FDG uptake may be augmented in high-grade, dedifferentiated, or metastatic castration-resistant prostate cancer (mCRPC), its sensitivity for detecting nodal metastases in the hormone-sensitive setting is disappointingly low. Compounding this limitation, the non-specific nature of ^18^F-FDG as a metabolic imaging agent predisposes to false-positive uptake in inflammatory, infectious, or granulomatous processes, consequently impairing diagnostic specificity—particularly in the assessment of pelvic lymph nodes where discrimination from benign pathological conditions is imperative (14).

Prostate-specific membrane antigen (PSMA), a type-II transmembrane glycoprotein, is markedly over-expressed in virtually all prostate carcinomas, with low background expression in most normal tissues (15). After conjugation to the macrocyclic chelator DOTA (1,4,7,10-tetraazacyclododecane-1,4,7,10-tetra-acetic acid) and radiolabeling with ^68^Ga, the resulting radiopharmaceutical ^68^Ga-PSMA-11 targets the marked overexpression of PSMA on prostate cancer cells. This enables ^68^Ga-PSMA-11 PET/CT to emerge as a highly sensitive and specific modality for primary tumor visualization, lymph node staging and detection of distant metastases, often outperforming ^18^F-FDG (16–18).

Although several head-to-head studies have compared ^18^F-FDG and ^68^Ga-PSMA-11 PET/CT for primary staging, biochemical recurrence or castration-resistant prostate cancer (CRPC), comparative data specifically addressing post-therapeutic evaluation of residual disease remain scarce. The present investigation was therefore designed to compare the performance of ^18^F-FDG and ^68^Ga-PSMA-11 PET/CT in detecting residual tumor burden after systemic therapy in patients with metastatic prostate cancer.

## Materials and methods

### Patients

This retrospective study included 26 consecutive patients with histologically confirmed *de novo* metastatic prostate cancer who underwent both ^68^Ga-PSMA-11 and ^18^F-FDG PET/CT after completing first-line local and/or systemic therapy at Nanyang Central Hospital between January 2023 and June 2025. Clinical, imaging and histopathological data were extracted from the institutional database. Dual-tracer PET/CT with both ^18^F-FDG and ^68^Ga-PSMA-11 was performed based on specific clinical indications rather than routine protocol. The decision to utilize both imaging modalities was made by the multidisciplinary tumor board (urology, oncology, and nuclear medicine) in the following circumstances: (1) patients with suspected disease progression despite rising PSA but inconclusive findings on conventional imaging; (2) evaluation of treatment response after systemic therapy when residual disease extent needed precise delineation for salvage treatment planning; or (3) discordance between PSA levels and morphological imaging findings requiring comprehensive metabolic characterization. This selective approach reflects real-world clinical decision-making in complex metastatic prostate cancer cases where single-modality imaging was deemed insufficient for accurate restaging. Inclusion criteria: (1) Histologically proven prostate cancer with distant metastases confirmed by conventional imaging at initial diagnosis (i.e., *de novo* metastatic disease), defined as at least one of the following: (a) bone metastases identified on whole-body bone scintigraphy (^99m^Tc-MDP) with correlation on CT or MRI; (b) lymph node metastases measuring ≥1.0 cm in short axis on contrast-enhanced CT (ceCT) or MRI with morphological features of malignancy (round shape, loss of fatty hilum, or necrosis); or (c) visceral metastases (liver, lung, or other organs) confirmed on ceCT or MRI with characteristic imaging features. PET/CT findings alone were not sufficient for initial metastasis diagnosis to avoid incorporation bias; (2) Completion of first-line local and/or systemic anticancer therapy (including androgen deprivation therapy, radical prostatectomy, radiotherapy, chemotherapy, or combination regimens) with subsequent restaging requirement; (3) Both PET/CT studies performed within a 7-day interval. Exclusion criteria: (1) Absence of prior treatment; (2) Incomplete imaging or clinical records. Median serum PSA at the time of ^68^Ga-PSMA-11 PET/CT was 26.53 ng/ml (range 0.45–776.0 ng/ml). Detailed patient characteristics, including prior treatment modalities, are summarized in **Supplementary Table**.

### Acquisition of PET/CT images

#### ^18^F-FDG PET/CT

Patients fasted for 4–6 hours and blood glucose was verified to be 3.9–6.1 mmol/L immediately before tracer injection. ^18^F-FDG was administered intravenously at a dose of 3.7 MBq/kg (range: 259–444 MBq for 70-kg patient). Patients were instructed to void urine immediately prior to imaging. PET/CT acquisition was performed 45–60 minutes post-injection using a Siemens Biograph mCT Flow 64-slice PET/CT scanner (Siemens Healthineers, Erlangen, Germany) or equivalent.

Scan range: vertex to mid-thigh (skull base to upper femora). CT acquisition: 120 kV, 120 mA (CareDose 4D automated exposure control), 3.0 mm slice thickness, 5 mm reconstruction interval, pitch 0.813, rotation time 0.5 s. PET acquisition: 3-dimensional (3D) mode, 2–3 minutes per bed position, 6–7 bed positions per scan. Images were reconstructed using ordered-subsets expectation maximization (OSEM) algorithm (4 iterations, 21 subsets) with Gaussian filtering (5 mm full width at half maximum) and CT-based attenuation correction.

#### ^68^Ga-PSMA-11 PET/CT

No specific dietary preparation was required. Patients were instructed to void urine before tracer administration and again immediately before imaging to minimize urinary excretion artifacts. ^68^Ga-PSMA-11 was synthesized using a ^68^Ge/^68^Ga generator (Eckert & Ziegler, Germany or ITG, Germany) and administered intravenously at 1.85 MBq/kg (range: 130–222 MBq for 70-kg patient). PET/CT acquisition was performed 45–60 minutes post-injection using the same scanner and identical acquisition parameters as ^18^F-FDG PET/CT, including vertex-to-mid-thigh scan range, 2–3 minutes per bed position, and identical reconstruction algorithms.

All acquisition parameters were established in accordance with the joint EANM/SNMMI procedure guideline for PSMA-ligand PET/CT imaging and standardized ^18^F-FDG PET/CT protocols (19, 20).

### Image analysis

All PET/CT datasets were independently reviewed in a blinded fashion by two board-certified nuclear-medicine physicians with >10 years of experience. Discrepancies were resolved by consensus.

Lesion evaluation combined visual assessment with semi-quantitative analysis. Spherical volumes of interest (VOIs) were manually drawn over every suspected lesion on both ^68^Ga-PSMA-11 and ^18^F-FDG images to obtain the maximum standardized uptake value (SUVmax). Background activity was defined as the mean SUVmax of gluteal skeletal muscle, a reference region widely adopted in previous PET/CT studies for tumor-to-background ratio calculation due to its low and stable tracer uptake (21). For osseous lesions, the SUVmax of the contralateral femoral head—or an adjacent non-involved bone when the contralateral side was also affected—was used as background, in accordance with previously validated methodologies (22). The tumor-to-background ratio (TBR) was calculated as: TBR = lesion SUVmax ÷ background SUVmax, a metric extensively validated in phantom and clinical PSMA PET/CT studies (23).

### Reference standard

Although the initial diagnosis of prostate cancer was histologically confirmed in all patients, systematic biopsy of each PET/CT-positive lesion was not feasible due to ethical and technical limitations. Therefore, a composite reference standard incorporating follow-up cross-sectional imaging and clinical/laboratory findings was adopted.

All patients underwent contrast-enhanced CT (ceCT) or MRI within 14 days after the completion of both PET/CT scans, and prior to any subsequent line of systemic therapy. This initial follow-up imaging served as a new morphological baseline for subsequent comparisons.

Thereafter, imaging follow-up was repeated at 3-month intervals. Lesions were classified as malignant if they met any of the following criteria:

1. progression according to RECIST 1.1 (≥20% increase in sum of diameters or appearance of new lesions) on at least two consecutive follow-up examinations;
2. unequivocal regression (≥30% decrease in size) in response to ongoing therapy, confirmed on at least two consecutive follow-up examinations; or
3. histopathologically proven when biopsy was clinically indicated.

Lesions that remained stable in size on ≥6 months of follow-up imaging, without corresponding PSA progression or clinical deterioration, were considered benign.

In addition, serial serum PSA levels were reviewed and correlated with imaging findings; a confirmed PSA decline of ≥50% from baseline was considered supportive of treatment response, while a rise of ≥25% was considered suggestive of progression.

To minimize verification bias, all follow-up images were interpreted by a radiologist blinded to the PET/CT results.

### Statistical analysis

All analyses were performed with SPSS 17.0 (IBM, Armonk, NY) and R version 4.2.0 (R Foundation for Statistical Computing). Normality of paired differences was assessed using the Shapiro-Wilk test. For normally distributed differences, paired t-tests were used; for non-normal distributions, Wilcoxon signed-rank tests were applied. To address clustering of multiple lesions within patients, lymph node metastases were analyzed at the patient level by averaging SUVmax and TBR values per patient. Bonferroni correction was applied for multiple comparisons across the three lesion categories (primary tumor, bone metastases, lymph nodes), setting the significance threshold at P < 0.017 (0.05/3). Effect sizes were reported as Cohen's d for t-tests and rank-biserial correlation for Wilcoxon tests. A two-tailed P-value < 0.05 was considered statistically significant.

## Results

### Patient characteristics

Twenty-six treated prostate cancer patients (mean age 70.1 ± 7.9 years, range 52–81) were enrolled. All tolerated ^68^Ga-PSMA-11 without adverse events. Every patient completed at least 6 months of contrast-enhanced CT or MRI follow-up (median, 8.5 months; range, 6–14 months), enabling longitudinal assessment of lesion stability or progression.

### Lesion detection in primary tumor

Among the 26 patients, ^68^Ga-PSMA-11 PET/CT visualized residual primary prostate tumors in all 26 cases (100%), whereas ^18^F-FDG PET/CT detected residual disease in 24 of 26 patients (92%). The SUVmax range of the ^18^F-FDG PET/CT group was 2.62 to 14.08, and the TBR range was 2.50 to 13.42. The SUVmax range of the ^68^Ga-PSMA-11 PET/CT group was 4.57 to 73.11, and the TBR range was 7.22 to 115.50. We further compared the SUVmax and TBR between the two imaging agents and found that ^68^Ga-PSMA-11 had higher SUVmax and TBR on the primary tumors of prostate cancer patients after diagnosis and treatment than ^18^F-FDG (median 17.04 (10.21-24.86) vs 5.05 (3.90-6.38), P < 0.001, r = 0.81; median 26.91 (16.13-39.26) vs 4.81 (3.72-6.08), P < 0.001, r = 0.85) (**Table 1**).

**Table 1:** Comparison of SUVmax and TBR between ^18^F-FDG and ^68^Ga-PSMA-11 PET/CT in primary prostate tumor and bone metastases.

Lesion site	Parameter	^18^F-FDG	^68^Ga-PSMA-11	P-value	Effect size	Statistical method
Primary tumor (n=24)	SUVmax	5.05 (3.90–6.38)	17.04 (10.21–24.86)	<0.001*	r = 0.81	Wilcoxon signed-rank test
	TBR	4.81 (3.72–6.08)	26.91 (16.13–39.26)	<0.001*	r = 0.85	Wilcoxon signed-rank test
Bone metastases (n=18)	SUVmax	8.29 ± 5.02	33.77 ± 23.51	<0.001*	d = 0.95	Paired t-test
	TBR	6.56 ± 3.97	33.08 ± 23.03	<0.001*	d = 1.03	Paired t-test

Data are presented as median (IQR) for non-normal distributions or mean ± SD for normal distributions. *P < 0.017 after Bonferroni correction for 3 lesion categories (α = 0.05/3). r = rank-biserial correlation coefficient; d = Cohen's d.

### Lesion detection in bone metastases

Nineteen patients had ^68^Ga-PSMA-11–positive osseous lesions, predominantly in the pelvis, axial skeleton and long bones. Per-patient detection rates: 95 % (18/19) with ^18^F-FDG vs 100 % (19/19) with ^68^Ga-PSMA-11. Mean SUVmax and TBR were again markedly higher for ^68^Ga-PSMA-11 (33.77 ± 23.51 and 33.08 ± 23.03) than for ^18^F-FDG (8.29 ± 5.02 and 6.56 ± 3.97; both P < 0.001, d = 0.95 and d = 1.03, respectively) (**Table 1**).

### Lesion detection in lymph node metastases

**Figure 1:**
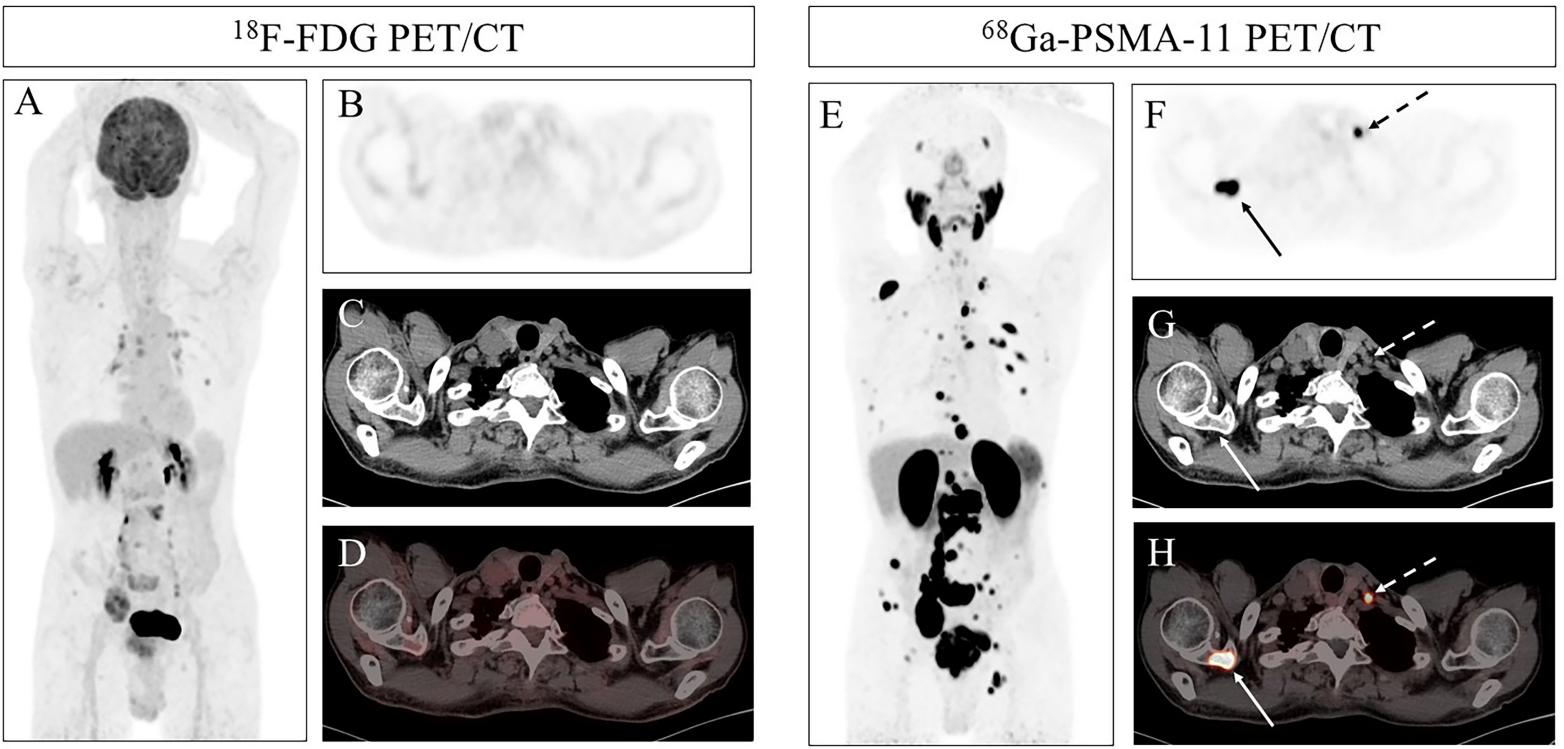
(Patient 14) A 69-year-old man presented with a 2-year history of weak stream and 2-month history of lower-back pain. Pelvic MRI suggested prostate cancer. ^18^F-FDG PET/CT Maximum-intensity-projection (MIP) and axial images **(A, B)** showed no hypermetabolic foci in the left supraclavicular region. Concomitant CT and fused PET/CT slices **(C, D)** confirmed absent FDG uptake in this area. ^68^Ga-PSMA-11 PET/CT MIP and axial views **(E, F)** revealed intense PSMA expression in a left supraclavicular lymph node (dashed arrow; SUVmax 14.81) and in the right scapula (solid arrow; SUVmax 41.39). Furthermore, ^68^Ga-PSMA-11 PET/CT MIP showed additional bone metastasis lesions compared to ^18^F-FDG PET/CT. CT and fused images **(G, H)** precisely localized both lesions, which were subsequently verified as metastatic on follow-up imaging.

**Figure 2:**
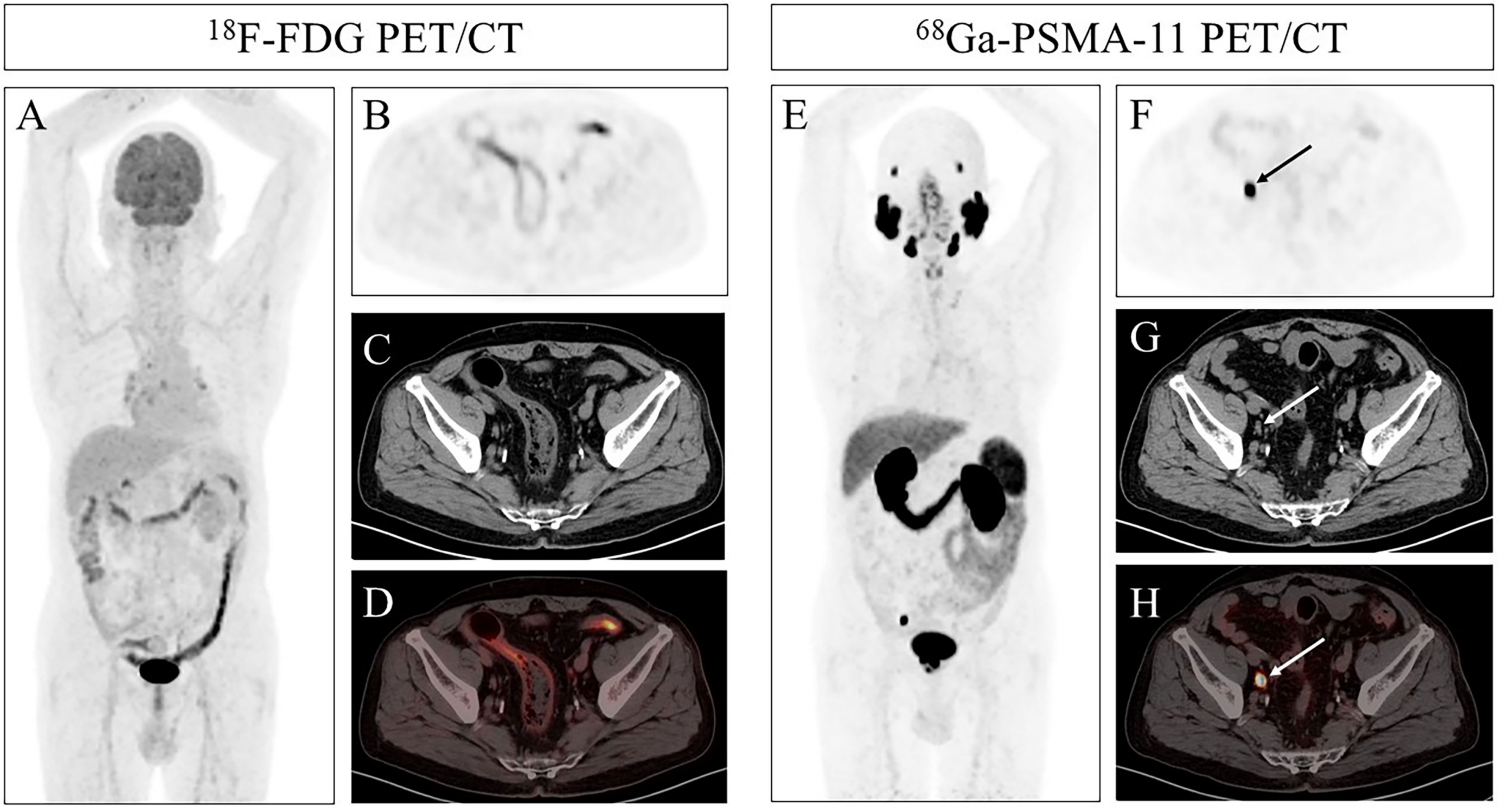
(Patient 21) An 80-year-old man presented with a 2-year history of progressive voiding difficulty and a serum PSA of 30.70 ng/ml. ^18^F-FDG PET/CT MIP and axial images **(A, B)** showed no abnormal FDG uptake in the right external iliac region. Corresponding CT and fused PET/CT slices **(C, D)** confirmed the absence of hypermetabolic lymphadenopathy. ^68^Ga-PSMA-11 PET/CT MIP and axial views **(E, F)** revealed intense PSMA avidity in a right external iliac lymph node (solid arrow; SUVmax 15.51). **(G, H)** CT and fused PET/CT images precisely localized the right external iliac lymph node metastasis, which was validated by follow-up imaging. Multiple lymph nodes, the left lobe of the liver, and the splenic parenchyma showed no corresponding increase in FDG uptake (black arrow).

Among the 26 patients, 17 had lymph node metastases confirmed by the composite reference standard. ^68^Ga-PSMA-11 PET/CT correctly identified metastatic lymph nodes in all 17 patients (100%). In contrast, ^18^F-FDG PET/CT showed abnormal uptake in 21 patients; however, only 7 of these patients had true-positive findings, while the remaining 14 patients exhibited false-positive lymph node uptake without corresponding metastases on follow-up imaging or histopathology (2 by postoperative histopathology, 15 by follow-up imaging). A total of 54 metastatic lymph nodes were identified in these 17 patients. The per-lesion detection rate was 91% (49/54) for ^18^F-FDG PET/CT and 96% (52/54) for ^68^Ga-PSMA-11 PET/CT. ^68^Ga-PSMA-11 detected all 52 lesions identified by either tracer, including 3 metastatic deposits in the left lower hilum, left supraclavicular area, and right external iliac region that were missed by ^18^F-FDG PET/CT (**Figures 1**, **2**). When comparing the 52 metastatic lymph nodes detected by the two tracers, ^68^Ga-PSMA-11 PET/CT had higher SUVmax and TBR than ^18^F-FDG PET/CT (17.02 (9.40-27.07) vs 4.17 (3.55-5.17), P = 0.005, r = 0.80; 26.88 (14.85-42.77) vs 3.96 (3.38-4.93), P = 0.002, r = 0.85) (**Table 2**). Among the 17 patients with confirmed lymph node metastases, ^18^F-FDG PET/CT detected 49 of the 52 true metastatic nodes (94%) identified by ^68^Ga-PSMA-11. In addition, ^18^F-FDG PET/CT showed abnormal uptake in 31 false-positive lymph nodes across 14 patients without corresponding metastases. These false-positive sites were most frequently observed in the mediastinum, bilateral hila, inguinal, submandibular, carotid sheath, and external iliac regions. The mean SUVmax of these false-positive nodes was 5.53 ± 3.11 (range up to 14.73), and mean TBR was 5.19 ± 2.92 (range up to 14.04). None of these nodes showed PSMA avidity on ^68^Ga-PSMA-11 PET/CT. In this patient (**Figure 2**), no abnormal FDG uptake was observed in multiple lymph nodes, the left hepatic lobe, or the splenic parenchyma, and ^68^Ga-PSMA-11 PET/CT showed no corresponding PSMA-avid lesions at these sites, further supporting the absence of visceral or nodal involvement.

**Table 2:** Comparison of SUVmax and TBR between ^18^F-FDG and ^68^Ga-PSMA-11 PET/CT in lymph node metastases (patient-level analysis).

Parameter	^18^F-FDG	^68^Ga-PSMA-11	P-value	Effect size
SUVmax	4.17 (3.55–5.17)	17.02 (9.40–27.07)	0.005*	r = 0.80
TBR	3.96 (3.38–4.93)	26.88 (14.85–42.77)	0.002*	r = 0.85

Data are median (IQR). n = 14 patient pairs (17 patients with lymph node metastases; 3 excluded due to missing FDG data). Patient-level analysis performed to address clustering (original: 17 patients, 54 lesions; range 1–12 lesions per patient). * P < 0.017 after Bonferroni correction. Wilcoxon signed-rank test used (non-normal distribution).

## Discussion

Accurate identification of recurrent disease, nodal involvement, and bone metastases is essential for guiding salvage therapy and predicting prognosis in metastatic prostate cancer. ^68^Ga-PSMA-11 PET/CT combines high tumor-to-background contrast with improved lesion detectability for lesions expressing PSMA, and has been shown to influence post-therapeutic decision-making (24). In the present cohort, ^68^Ga-PSMA-11 consistently yielded higher SUVmax and TBR than ^18^F-FDG for the treated prostate bed, lymph node and bone metastases. Nevertheless, PSMA-avid benign lesions, such as those arising from benign prostatic hyperplasia or active prostatitis, may lead to false-positive interpretations, underscoring the importance of correlation with anatomic imaging and confirmatory follow-up (25).

For lymph node staging, ^68^Ga-PSMA-11 identified three additional metastatic deposits that were overlooked by ^18^F-FDG, while simultaneously excluding 31 FDG-positive but clinically irrelevant nodes. This discriminatory power reduces unnecessary invasive biopsies and may refine radiation-field planning. Similarly, one patient with occult bone metastases was detected only by ^68^Ga-PSMA-11, underscoring the added value in skeletal survey.

Conversely, two histologically confirmed nodal metastases were FDG-avid but PSMA-negative, suggesting a possible dedifferentiated phenotype. Down-regulation of PSMA expression after androgen-deprivation or chemotherapy, together with emergence of dedifferentiated, glycolytic clones, may explain this discordance (26). Thus, a dual-tracer strategy—rather than a simple substitution—could improve sensitivity across the biologic spectrum of treated disease.

The study cohort was heterogeneous in terms of prior treatments—including radical prostatectomy, radiotherapy, ADT, and chemotherapy—which may confound tracer uptake comparisons, as these modalities differentially modulate PSMA expression and glucose metabolism (**Table 3**). ADT can induce complex changes in tracer uptake: while chronic ADT may downregulate PSMA expression in some patients, short-term ADT can cause PSMA flare with paradoxically increased uptake (27). Similarly, ADT may increase FDG uptake through metabolic reprogramming toward glycolysis (28). Chemotherapy-induced cellular stress and dedifferentiation can also alter both PSMA expression and glucose metabolism, leading to discordant tracer uptake patterns. Radiotherapy may cause inflammatory changes that increase non-specific FDG uptake while potentially affecting PSMA expression in irradiated fields (29). These treatment-related biological variations likely contributed to the observed inter-patient variability in SUVmax and TBR values, and may explain why certain lesions were detected by one tracer but not the other (30). Future studies should stratify analyses by treatment history or focus on homogeneously treated cohorts to better delineate the specific impact of prior therapy on dual-tracer performance.

**Table 3:** Characteristics of patients (PSA, Prostate-specific antigen; ADT, Androgen deprivation therapy).

Patient	age	Gleason score at the initial diagnosis	PSA (ng/ml)	Radical prostatectomy	External radiotherapy of the prostate	Radiotherapy of the prostate bed	Chemotherapy before PET	ADT before PET
1	75	7	15.93	+	–	+	–	+
2	77	9	510.54	–	–	–	+	+
3	63	8	26.53	–	+	–	–	–
4	59	8	5.74	+	+	–	–	–
5	74	8	3.51	–	+	+	+	–
6	70	9	776.00	+	–	+	+	+
7	75	9	263.84	+	–	+	–	–
8	81	7	1.98	+	–	–	+	+
9	57	8	15.94	+	+	+	–	–
10	77	7	258.91	–	+	–	–	–
11	81	9	746.34	–	–	–	+	–
12	79	7	28.11	+	+	+	+	+
13	68	9	603.15	–	+	+	–	–
14	69	9	4.02	+	–	+	+	+
15	73	8	2.36	+	–	–	+	–
16	71	9	21.94	–	+	–	–	+
17	67	8	563.28	–	–	+	+	–
18	66	9	405.44	+	–	–	–	–
19	58	9	8.06	–	+	+	+	+
20	65	7	41.27	+	+	+	+	–
21	80	9	30.70	–	–	+	+	–
22	64	9	26.53	+	–	+	–	+
23	75	8	0.45	+	+	–	+	–
24	52	8	3.72	+	–	+	+	+
25	69	9	273.19	–	+	–	+	+
26	78	7	7.33	+	+	+	–	–

In addition to the comparative analysis of tracer performance, we explored potential associations between clinicopathological variables (Gleason score and serum PSA) and PET/CT parameters. Although this study was not primarily designed to establish such correlations, we observed that higher Gleason scores (≥8) tended to be associated with elevated SUVmax and TBR values on both ^68^Ga-PSMA-11 and ^18^F-FDG PET/CT, particularly in primary tumors and bone metastases. However, no statistically significant linear correlation was found between PSA levels and SUVmax or TBR in this cohort, possibly due to the heterogeneous treatment histories and the small sample size. Future studies with larger, treatment-naive or uniformly treated cohorts are warranted to validate these preliminary observations and to elucidate the biological underpinnings of tracer uptake in relation to tumor grade and serum biomarkers.

Several limitations of this study should be acknowledged. First, the sample size was modest (n=26), and the study was conducted at a single institution, limiting generalizability. Second, we report lesion-based detection rates rather than formal diagnostic accuracy metrics (sensitivity, specificity, positive/negative predictive values with 95% confidence intervals) against a histopathological gold standard. Systematic biopsy of all suspected lesions was not feasible for ethical and technical reasons; instead, we employed a composite reference standard incorporating longitudinal imaging follow-up, which may introduce verification bias. Consequently, our findings should be considered exploratory and hypothesis-generating rather than definitive evidence of diagnostic superiority. Third, as noted above, the heterogeneity of prior treatments (surgery, radiotherapy, chemotherapy, ADT) and variable treatment intervals precluded stratified analyses to isolate treatment-specific effects on tracer performance. Fourth, the interval between therapy completion and PET/CT imaging varied among patients, potentially affecting the biological behavior of residual disease and tracer uptake characteristics. Finally, the retrospective design and selective referral of complex cases to dual-tracer imaging may have introduced selection bias. Multi-institutional prospective studies with larger, homogeneously treated cohorts and standardized reference standards are warranted to validate these preliminary observations and establish formal diagnostic accuracy metrics.

## Conclusion

^68^Ga-PSMA-11 PET/CT demonstrates higher tracer avidity and tumor-to-background contrast than ^18^F-FDG PET/CT for identifying residual primary tumor, nodal and osseous disease after therapy in metastatic prostate cancer. However, ^18^F-FDG PET/CT may capture metabolically active but PSMA-negative lesions in a subset of treated patients. A complementary, dual-tracer approach is therefore recommended to improve the detection of post-therapeutic residual disease and to guide subsequent clinical management.

## Data Availability

The original contributions presented in the study are included in the article/**Supplementary Material**. Further inquiries can be directed to the corresponding author.
