# Supplementary material for: Differences in residual lesion detection after treatment of metastatic prostate cancer based on dual-tracer PET/CT
Source: Front Oncol. 2026 Mar 12;16:1713930. doi: 10.3389/fonc.2026.1713930 (PMC13019480; doi:10.3389/fonc.2026.1713930)
Supplement: Supplementary file 1 [file DataSheet1.docx]

| Variable | W statistic | P-value | Distribution | Test selected |
| --- | --- | --- | --- | --- |
| Primary tumor SUVmax (difference) | 0.865 | 0.004 | Non-normal | Wilcoxon |
| Primary tumor TBR (difference) | 0.856 | 0.003 | Non-normal | Wilcoxon |
| Bone metastases SUVmax (difference) | 0.924 | 0.155 | Normal | Paired t-test |
| Bone metastases TBR (difference) | 0.919 | 0.122 | Normal | Paired t-test |
| Lymph node SUVmax (difference) | 0.861 | 0.032 | Non-normal | Wilcoxon |
| Lymph node TBR (difference) | 0.855 | 0.026 | Non-normal | Wilcoxon |

**Supplement Table S1** Shapiro-Wilk Normality Testing of Paired Differences for SUVmax and TBR

**Supplement Table S2** Sensitivity Analysis: Comparison of Patient-Level and Lesion-Level Approaches for Lymph Node Metastases

| Analysis approach | n | Mean FDG | Mean PSMA | Test | P-value | Significant* |
| --- | --- | --- | --- | --- | --- | --- |
| Patient-level (correct) | 14 | 4.17 ± 2.65 | 17.02 ± 14.35 | Wilcoxon | 0.005 | Yes |
| Lesion-level (incorrect) | 50 | 5.77 ± 3.92 | 20.77 ± 18.43 | t-test | <0.001 | Yes |

At α = 0.017 after Bonferroni correction.

**Supplement Table S3** Comparison of SUVmax and TBR of two agents in primary prostate tumor and/or bone metastases

|  | Primary Prostate Tumor | | | | Bone Metastases | | | |
| --- | --- | --- | --- | --- | --- | --- | --- | --- |
| Patient | SUVmax (FDG) | SUVmax (PSMA) | TBR (FDG) | TBR (PSMA) | SUVmax (FDG) | SUVmax (PSMA) | TBR (FDG) | TBR (PSMA) |
| 1 | - | 14.23 | - | 22.48 | 5.93 | 64.05 | 4.69 | 62.73 |
| 2 | 8.81 | 6.03 | 8.40 | 9.53 | 8.50 | 20.28 | 6.72 | 19.86 |
| 3 | 3.71 | 51.46 | 3.54 | 81.30 | 4.13 | 25.00 | 3.27 | 24.49 |
| 4 | 5.86 | 18.78 | 5.59 | 29.67 | - | - | - | - |
| 5 | 3.63 | 34.21 | 3.46 | 54.04 | 5.21 | 64.38 | 4.12 | 63.06 |
| 6 | 3.22 | 6.45 | 3.07 | 10.19 | - | - | - | - |
| 7 | 4.53 | 15.29 | 4.32 | 24.15 | - | 4.61 | - | 4.52 |
| 8 | 11.50 | 19.51 | 10.96 | 30.82 | 5.49 | 61.50 | 4.34 | 60.24 |
| 9 | 3.93 | 14.11 | 3.75 | 22.29 | 21.68 | 8.12 | 17.15 | 7.95 |
| 10 | 2.62 | 5.76 | 2.50 | 9.10 | 5.03 | 7.88 | 3.98 | 7.72 |
| 11 | - | 7.85 | - | 12.40 | - | - | - | - |
| 12 | 5.86 | 24.67 | 5.59 | 38.97 | 8.03 | 16.01 | 6.35 | 15.68 |
| 13 | 9.13 | 10.76 | 8.70 | 17.00 | 11.89 | 15.04 | 9.41 | 14.73 |
| 14 | 6.83 | 21.51 | 6.51 | 33.98 | 7.86 | 41.39 | 6.22 | 40.54 |
| 15 | 5.55 | 42.91 | 5.29 | 67.79 | - | - | - | - |
| 16 | 4.72 | 35.83 | 4.50 | 56.60 | 5.83 | 56.10 | 4.61 | 54.95 |
| 17 | 5.38 | 8.56 | 5.13 | 13.52 | 5.10 | 20.16 | 4.03 | 19.75 |
| 18 | 6.23 | 25.41 | 5.94 | 40.14 | 6.22 | 25.38 | 4.92 | 24.86 |
| 19 | 6.03 | 15.12 | 5.75 | 23.89 | 17.21 | 40.11 | 13.62 | 39.29 |
| 20 | 4.64 | 73.11 | 4.42 | 115.50 | 2.33 | 91.28 | 1.84 | 89.40 |
| 21 | 3.81 | 21.40 | 3.63 | 33.81 | - | - | - | - |
| 22 | 4.36 | 14.09 | 4.16 | 22.26 | 6.67 | 23.26 | 5.28 | 22.78 |
| 23 | 7.38 | 5.16 | 7.04 | 8.15 | - | - | - | - |
| 24 | 4.12 | 4.57 | 3.93 | 7.22 | - | - | - | - |
| 25 | 14.08 | 18.78 | 13.42 | 29.67 | 16.38 | 10.89 | 12.96 | 10.67 |
| 26 | 3.41 | 17.08 | 3.25 | 26.98 | 5.69 | 17.08 | 4.50 | 16.73 |

|  | |  | | Lymph Node Metastases | | | | FDG-positive PSMA-negative Lymph Nodes | | | |
| --- | --- | --- | --- | --- | --- | --- | --- | --- | --- | --- | --- |
| patient | No. of lesions | | SUVmax (FDG) | | SUVmax (PSMA) | TBR (FDG) | TBR  (PSMA) | No. of lesions | SUVmax (FDG) | TBR  (FDG) |  |
| 1 | 1 | | 2.32 | | 10.56 | 2.20 | 16.68 | 3 | 3.63±2.12 | 5.73±3.35 |  |
| 2 | 1 | | 3.61 | | 2.82 | 3.40 | 4.45 | 2 | 3.86±1.12 | 3.68±1.07 |  |
| 3 | 2 | | - | | 24.53±9.72 | - | 38.75±15.36 | 1 | 14.73 | 14.04 |  |
| 4 | 0 | | - | | - | - | - | 6 | 3.63±0.81 | 3.46±0.77 |  |
| 5 | 9 | | 2.41±0.19 | | 59.53±16.67 | 2.30±0.18 | 94.04±26.33 | 0 | - | - |  |
| 6 | 0 | | - | | - | - | - | 4 | 3.60±1.52 | 3.43±1.45 |  |
| 7 | 12 | | 10.28±7.29 | | 5.10±1.78 | 9.80±6.95 | 8.06±2.81 | 1 | 2.58 | 2.46 |  |
| 8 | 4 | | 9.41±5.43 | | 20.22±4.16 | 8.97±5.18 | 31.94±6.57 | 0 | - | - |  |
| 9 | 0 | | - | | - | - | - | 1 | 3.90 | 3.70 |  |
| 10 | 0 | | - | | - | - | - | 1 | 14.10 | 13.40 |  |
| 11 | 0 | | - | | - | - | - | 2 | 7.28±0.63 | 6.94±0.60 |  |
| 12 | 0 | | - | | - | - | - | 1 | 6.30 | 6.00 |  |
| 13 | 0 | | - | | - | - | - | 2 | 7.42±3.81 | 7.07±3.63 |  |
| 14 | 3 | | 5.22±3.74 | | 22.38±10.75 | 4.98±3.57 | 35.36±16.98 | 1 | 5.14 | 4.90 |  |
| 15 | 1 | | 4.04 | | 51.12 | 3.90 | 80.76 | 1 | 5.39 | 5.14 |  |
| 16 | 1 | | 4.11 | | 36.68 | 3.90 | 57.95 | 3 | 6.93±0.11 | 6.61±0.10 |  |
| 17 | 5 | | 3.53±0.81 | | 13.81±2.42 | 3.37±0.77 | 21.82±3.82 | 0 | - | - |  |
| 18 | 8 | | 5.01±0.73 | | 27.92±10.31 | 4.78±0.70 | 44.11±16.29 | 1 | 5.49 | 5.23 |  |
| 19 | 1 | | 3.42 | | 7.54 | 3.27 | 11.91 | 1 | 4.13 | 3.94 |  |
| 20 | 2 | | 4.91±3.70 | | 9.26±6.34 | 4.68±3.53 | 14.63±10.02 | 0 | - | - |  |
| 21 | 1 | | - | | 15.51 | - | 24.50 | 1 | 4.54 | 4.33 |  |
| 22 | 0 | | - | | - | - | - | 1 | 7.63 | 7.27 |  |
| 23 | 0 | | - | | - | - | - | 3 | 4.09±2.14 | 3.90±2.04 |  |
| 24 | 1 | | 4.23 | | 9.82 | 4.03 | 15.51 | 1 | 3.68 | 3.51 |  |
| 25 | 1 | | 7.81 | | 5.79 | 7.45 | 9.15 | 1 | 3.43 | 3.27 |  |
| 26 | 1 | | - | | 15.03 | - | 23.74 | 0 | - | - |  |

**Supplement Table S4** Comparison of SUVmax and TBR between the two tracers in lymph-node metastases and/or FDG-positive but PSMA-negative lymph nodes
